# Accelerating degradation rate of pure iron by zinc ion implantation

**DOI:** 10.1093/rb/rbw020

**Published:** 2016-06-05

**Authors:** Tao Huang, Yufeng Zheng, Yong Han

**Affiliations:** ^1^State Key Laboratory for Turbulence and Complex System and Department of Materials Science and Engineering, College of Engineering, Peking University, Beijing 100871, China; ^2^State Key Laboratory for Mechanical Behavior of Materials, Xian Jiaotong University, Xian 710049, China

**Keywords:** biodegradable metals, iron, zinc ion implantation, biocompatibility

## Abstract

Pure iron has been considered as a promising candidate for biodegradable implant applications. However, a faster degradation rate of pure iron is needed to meet the clinical requirement. In this work, metal vapor vacuum arc technology was adopted to implant zinc ions into the surface of pure iron. Results showed that the implantation depth of zinc ions was about 60 nm. The degradation rate of pure iron was found to be accelerated after zinc ion implantation. The cytotoxicity tests revealed that the implanted zinc ions brought a slight increase on cytotoxicity of the tested cells. In terms of hemocompatibility, the hemolysis of zinc ion implanted pure iron was lower than 2%. However, zinc ions might induce more adhered and activated platelets on the surface of pure iron. Overall, zinc ion implantation can be a feasible way to accelerate the degradation rate of pure iron for biodegradable applications.

## Introduction

Stents are widely used to treat vascular obstruction disease like atherosclerosis. Most of commercially available stents are made of corrosion resistant metals, such as stainless steels, Ni-Ti alloys, Co-Cr alloys and Pt-Ir alloys. The permanent remain of these stents in blood vessels may induce some serious side effects, including inflammatory reaction, medial atrophy, aneurysm formation, endometrial hyperplasia, and late in-stent restenosis [1].

To avoid these problems, the concept of biodegradable stents was put forward [2–4]. Iron-based materials [5–10] have been considered as promising candidates for biodegradable stent applications since their first safe implantation in rabbits for 1 year [11]. Subsequently, good biocompatibility and biosafety of pure iron for stent applications have been further verified [11, 12] by a series of both *in vitro* and *in vivo* tests. Nevertheless, the problems of too slow degradation rate and local corrosion mode significantly hampered the clinical utility of pure iron and need to be settled urgently. Many methods have been adopted to improve the corrosion behavior of pure iron, such as alloying [6, 13–20], compositing [21–25] and new manufacturing technics [26–28]. Many of these new developed iron based materials exhibited faster degradation than pure iron, but the corrosion rate still remained a great distance to the clinical requirements. Besides, surface modification has also been tried, including lanthanum ions implantation [29], deposited Fe-O films [30], plasma nitriding [31], Calcium zinc phosphate coating [32] and so forth. These modification methods effectively improved the biocompatibility of pure iron, while significantly increased the corrosion resistance.

In this work, metal vapor vacuum arc (MEVVA) was adopted to implant zinc ions into the surface of pure iron. Zn has been considered as a potential biodegradable metal which is being researched widely [33]. In addition, Zn is an indispensable component of numerous enzymes in the human body, which play important roles in regulating gene expression and keeping structural integrity. Besides, good antibacterial activity is also an important merit of zinc for biomaterial applications [34]. Due to the lower standard electrode potential (−0.7618 V) of zinc than that of iron (−0.44 V) [35], Fe-Zn solid solution is more susceptible to be corroded than pure iron [36]. In addition, the extra zinc ions that beyond solubility can cause serious distortion of iron lattice, then increase the system energy and decrease the corrosion potential of iron matrix.

## Materials and methods

### Material preparation

Zn ions were implanted into the surface of mechanically polished pure iron (purity, 99.9%) using a MEVVA ion source (Beijing Normal University, Beijing, China). The implantation parameters mainly included the extracted voltage of 45 kV, the vacuum level of 2 × 10^−3^ Pa, the implantation fluence of 2 × 10^17^ ions⋅cm^−2^, the beam current density of 2 mA⋅cm^−2^ and the maximum temperature of no more than 200 °C.

### Surface characterization

The elements as well as their distribution on the surface of zinc ion implanted pure iron were tested by an energy dispersive spectrometer (EDS) attached on an environmental scanning electronic microscope (ESEM, Quanta 200FEG). The depth profile was measured by Auger Electron Spectroscopy (AES, PHI-700, ULVAC-PHI, Japan) using SiO_2_ sputtering with the rate of 31 nm⋅min^−1^. The surface chemical composition was detected by X-ray photoelectron spectroscopy (XPS, Axis Ultra, KRATOS ANALYTICAL, Britain) using Al Kα radiation.

### Electrochemical tests

Electrochemical tests were carried out using an electrochemical work station (PGSTAT 302 N, Metrohm Autolab) with a saturated calomel electrode as reference electrode, a platinum electrode as the auxiliary electrode and the specimen as the working electrode. The electrolyte was Hank’s solution [37] with temperature of 37 ± 0.5°C and pH value of 7.4. The exposed area of the working electrode was 0.3318 cm^2^. The open circuit potential (OCP) was performed with time of 9000 s. Electrochemical impedance spectroscopy was tested between 100 kHz and 10 mHz. The potentiodynamic polarization curves were measured between (OCP value −600) mV and (OCP value 600) mV with the scanning rate of 0.33 mV⋅s^−1^. According to ASTM-G102-89 [38], corrosion rates based on electromechanical date could be calculated by the formulas below:

(1) $$V_{\text{corr}}=K_{1}\frac{I_{\text{corr}}}{\rho }\text{EW}$$

(2) $$\text{CR}=K_{2}I_{\text{corr}}\text{EW}$$

In these formulas, *V*_corr_ (mm⋅year^−1^), CR (mg⋅cm^−2^⋅day^−1^) and *EW* represent the penetration rate, weight loss rate and equivalent weight, respectively. *K_1_* and *K_2_* are constants with values of 3.27 × 10^−3^ mm⋅g⋅μA^−1^⋅cm^−1^⋅year^−1^ and 8.954 × 10^−3^ g⋅cm^2^⋅μA^−1^⋅m^−2^⋅day^−1^, respectively.

### Static immersion tests

In the *in vitro* static immersion tests, each specimen was soaked in 50 ml Hank’s solution with temperature of 37°C for 3, 15 and 30 days based on ASTM-G31-72 [39]. After immersion completed, specimens were gently rinsed by distilled water and quickly dried by a blower. The surface morphologies of specimens were observed under ESEM (Quanta 200FEG). The degradation rates based on weight loss were calculated using the following formula:

(3) $$\text{CR}=\frac{\Delta m}{St}$$

In this formula, CR (mg⋅cm^−2^⋅day^−1^), Δ*m* (mg), *S* (cm^2^) and *t* (day) represent the corrosion rate, the mass loss, the exposed surface area of the specimen to the solution and the immersion time, respectively.

### Cytotoxicity tests

Murine fibroblast cells (L-929), human umbilical vein endothelial cells (EA. hy-926) and human vascular smooth muscle cells (VSMC) were separately incubated in the Dulbecco’s modified Eagle’s medium (DMEM) with 100 U·ml^−1^ penicillin, 100 μg·ml^−1^ streptomycin and10% fetal bovine serum, in a humidified atmosphere with 5% CO_2_ at a temperature of 37°C. To prepare the extraction medium, specimens were soaked in serum-free DMEM with a surface area over medium volume ratio of 1.25 cm^2^⋅ml^−1^ in a humidified atmosphere with 5% CO_2_ at 37°C for 72 h according to ISO 10993-12 [40]. Then the obtained medium was centrifuged and the supernatant fluid was withdrawn as the extraction medium. Inductively coupled plasma atomic emission spectrometry (ICP-AES, Leeman, Profile) was adopted to measure the concentrations of metallic ions in the extraction medium. The selected cell lines were seeded in the 96-well plates with a density of about 5 × 10^3^ cells per 100 μl and cultured for 24 h to allow attachment. After that, the mediums were replaced by extraction medium, with DMEM as the negative control group and DMEM with 10% dimethyl sulfoxide as the positive control. 10 μl serum was added to each well. After 1, 2 and 4 days culture, 10 μl of cell counting kit solution was added to each well and put back to incubator for 3 h more culture. Thereafter, microplate reader (Bio-RAD680) was used to measure the absorbance at the wavelength of 450 nm. According to ISO 19003-5 [41], cells viability (*X*) was calculated by the following formula:

(4) $$X=\frac{{\text{OD}}_{1}}{{\text{OD}}_{2}}\times 100\%$$

In this formula, OD_1_ represents the average absorbance of extraction groups and positive control group. OD_2_ represents the average absorbance of negative control group.

### Hemolysis and platelet adhesion tests

The blood solution used in hemolysis tests was prepared by diluting healthy human blood (with 3.8 wt.% citric acid sodium) into physiological saline based on the volume ratio of 4:5. The specimen and 10 ml physiological saline were put into a centrifugal tube, with 10 ml physiological saline as the negative control and 10 ml distilled water as the positive control. All of these tubes were put into water bath and kept the temperature at 37°C for 0.5 h. After that, 0.2 ml of the prepared blood solution was added to each tube and remained the temperature at 37°C for 1 h. Then specimens were removed before centrifuging these tubes at 800 g for 5 min. The supernatant was withdrawn and put into 96-well plates. A microplate reader (Bio-RAD680) was adopted to measure the absorbance at the wavelength of 545 nm. Hemolysis was calculated according to the formula below:

(5) $$\text{Hemolysis}=\frac{\text{OD(test)}-\text{OD(negative control)}}{\text{OD(positive control)}-\text{OD(negative control)}}\times 100\%$$

In the platelet adhesion tests, the platelet rich plasma (PRP) was obtained from the supernatant after centrifuging healthy whole blood at the rate of 1000 r⋅min^−1^ for 10 min. Specimens were put into 24-well plates after sterilized by ultraviolet light. Then 0.2 ml PRP was dropped to cover the surface of the specimen. Then transfer the 24-well plates into the incubator with temperature of 37°C for 1 h. After that, the specimens were rinsed twice using phosphate buffered solution. The glutaraldehyde solution with concentration of 2.5% was adopted to fix the adhered platelets. ESEM (Quanta 200FEG) was used to observe the morphologies of adhered platelets.

## Results and discussion

### The composition and depth profiles

**Figure 1.**
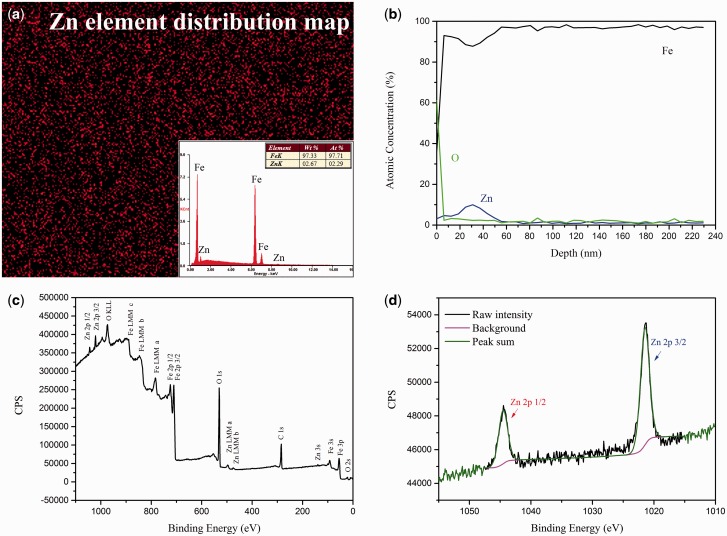
**(a)** EDS analysis, **(b)** AES measurements, **(c)** binding energy survey in the whole range and **(d)** high-resolution XPS spectra of Zn 2p.

The EDS results revealed that the Zn element uniformly distributed on the surface of pure iron with content of approximately 2.67 wt.% (Fig. 1a). The AES analysis results indicated that the implantation depth was about 60 nm and the highest zinc ions content of 11 at.% appeared at 30 nm in depth (Fig. 1b). The content distribution of zinc ions in depth matched Gaussion distribution [42–44]. On the other hand, the highest content of oxygen (over 60 at.%) was at the outmost surface, but it reduced quickly to only 5% at the depth of 7 nm. In contrast, the zinc content raised steadily between this depth range. The binding energy survey in the whole range of the specimen is illustrated in Figure 1c which revealed that the major elements on the surface layer of zinc ion implanted pure iron were Fe, Zn, O and C. Figure 1d shows the high-resolution XPS spectra on the binding state of Zn 2p. The binding energy of Zn 2p_3/2_ and Zn 2p_5/2_ were1044.3 and 1021.4 eV, respectively, which represented ZnO [45, 46]. Taking AES results into consideration, zinc element existed as ZnO in the outer layer with depth of about 7 nm. Afterwards, zinc would exist as atoms in Fe-Zn solid solution.

### Electrochemical corrosion behavior

**Figure 2.**
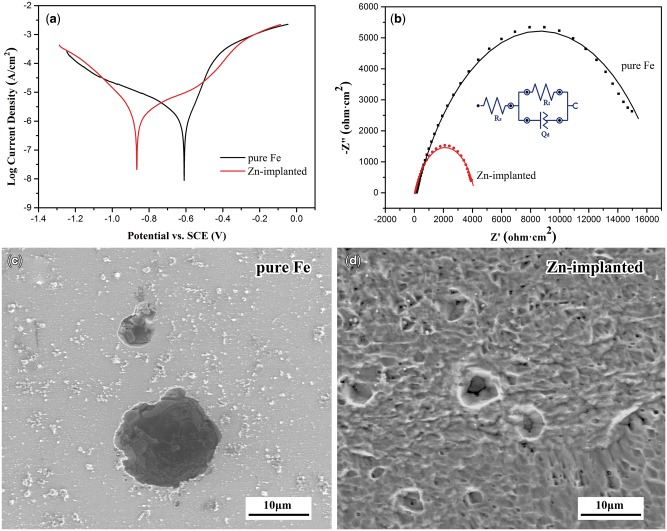
Electrochemical measurement results: **(a)** Tafel curves, **(b)** Nyquist plots, **(c)** and **(d)** are the surface morphologies of specimens after potentiodynamic polarization.

The electrochemical test results are shown in Figure 2. Figure 2a shows the Tafel curves, and the relative electrochemical parameters are displayed on Table 1. After implantation of zinc ions, the corrosion potential of pure iron decreased while the corrosion current density increased, revealing its higher corrosion tendency. The Nyquist plots are shown in Figure 2b with an equivalent circuit model [*R_s_*(*Q_d_R_t_*)] inserted. The parameters including *R_s_*, *Q_d_* and *R_t_* represent the electrolyte resistance, a constant phase element and the transfer resistance, and their values are listed on Table 2. The smaller transfer resistance means the faster corrosion rate [47]. Due to its much lower transfer resistance, zinc ion implanted pure iron exhibited faster corrosion than untreated pure iron.

**Table 1.** Average electrochemical parameters of Zn ion implanted pure iron (as-cast and as-sintered pure iron as control)

Materials	*E*_corr_ (V)	*I*_corr_ (μA. cm^−2^)	*V*_corr_ (mm/year)	Corrosion rate (mg. cm^−2^. d^−1^)	
				Electrochemical test	Immersion test for 30 days
Untreated pure iron	−0.582	2.340	0.027	0.059	0.048
Zn ion implanted pure iron	−0.901	8.001	0.093	0.213	0.060

Note: Corrosion potential (*E*_corr_), corrosion current density (*I*_corr_) and corrosion rate (*V*_corr_).

**Table 2.** Parameters for simulated circuit

Materials	*R*_s_ (Ω)	*R*_t_ (KΩ)	*Q*_d_ (μF)	*n*	*χ^2^*
Untreated pure iron	139	17	78.2	0.701	0.10925
Zn ion implanted pure iron	57.2	4.84	237	0.680	0.24493

The surface morphologies of samples after potentiodynamic polarization are shown in Figure 2c and d. The surface of pure iron remained nearly intact with a few corrosion pits. There was also localized corrosion found on the surface of zinc ions implanted pure iron, but much more corrosion pits were found and the size of pits was smaller. These results revealed that pure iron corroded faster and more uniform after implantation of zinc ions. The phenomenon might be attributed to the following reasons: on the one hand, the lower corrosion potential of zinc and the higher distortion energy after ion implantation endowed the implantation layer with a stronger corrosion tendency. On the other hand, the lower standard electrode potentials of Fe-Zn solid solutions and zinc atomic cluster than that of pure iron rendered the formation of galvanic corrosion between them and displayed accelerating corrosion effect on the implantation layer. Last but not the least, the uniform implantation of zinc ions on the surface layer of pure iron enabled the uniform distribution of galvanic cells [48]. Then the corrosion on the zinc ion implanted pure iron exhibited more uniform corrosion behavior than that of pure iron.

### Static immersion test

**Figure 3.**
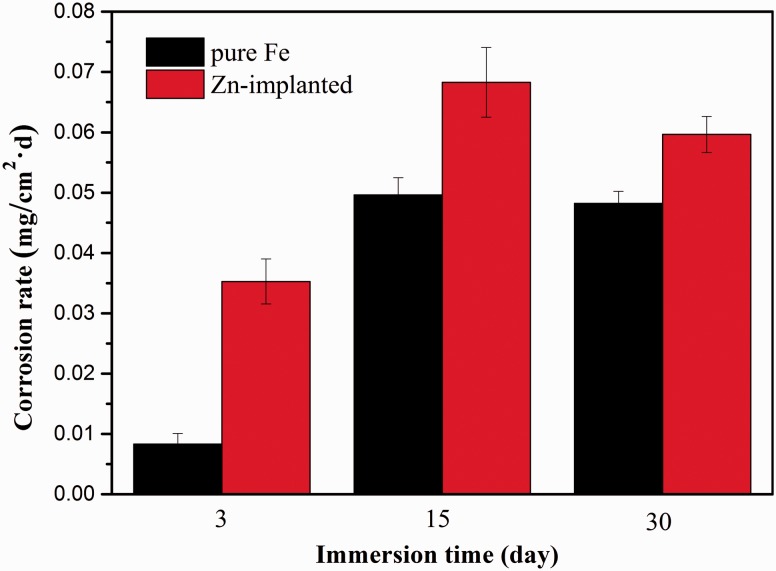
Corrosion rates based on weight loss after static immersion tests.

The corrosion rates based on the weight loss after static immersion tests are shown in Figure 3. During the whole test period, zinc ion implanted pure iron exhibited faster corrosion than the one without ion implantation, and its corrosion rate reached to nearly four times as that of untreated pure iron after 3 days immersion. Subsequently, the corrosion rate difference between these two groups reduced gradually.

**Figure 4.**
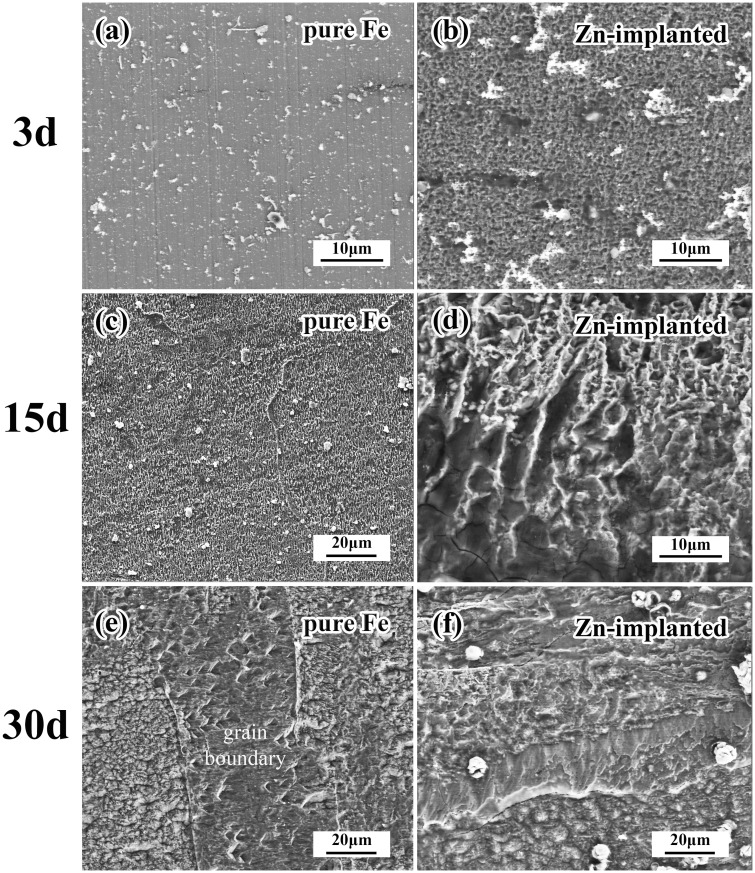
Surface morphologies of specimens after static immersion tests.

The surface morphologies of specimens after static immersion are shown in Figure 4. There was no significant change on the surface morphology of pure iron after 3 days test. However, uniform corrosion could be observed on the zinc ion implanted samples. The morphology revealed approximately uniform corrosion of pure iron with only a few deep corrosion pits. Comparing to the 3 days results, more serious and nonuniform corrosion behavior was observed on the zinc ion implanted samples after 15 days test. Experienced for 30 days test, grain boundary could be observed clearly on the surface of untreated pure iron, indicating a faster corrosion rate of grain boundary than that of the interior grain. It is worth mentioning that the zinc ion implanted pure iron corroded faster than untreated pure iron from the perspective of corrosion depth.

Because zinc has a certain solid solubility in iron [36], a part of implanted zinc ions were involved in the formation of Fe-Zn solid solution. The excess zinc ions beyond solubility were possible to get together to decrease the systematic energy. The Fe-Zn solid solution exhibited lower corrosion potential than pure iron [49]. In addition, Wang et al. [50] prepared three kinds of Fe-Zn alloys with different contents of Zn by electrochemical deposition, including Fe- 5 wt.% Zn, Fe- 10 wt.% Zn and Fe- 15 wt.% Zn alloys. The results showed that all of these three kinds of Fe-Zn alloys corroded faster than pure iron. Hence, the implantation of zinc ions into pure iron to form Fe-Zn solid solutions is a feasible way to speed up the corrosion of pure iron. On the other hand, the implantation of zinc ions can cause lattice distortion in the implantation layer. The corrosion in this area can also be accelerated as the high distortion energy decreases the corrosion potential. Base on above mentioned reasons, zinc ions implanted pure iron can corrode faster than pure iron.

**Figure 5.**
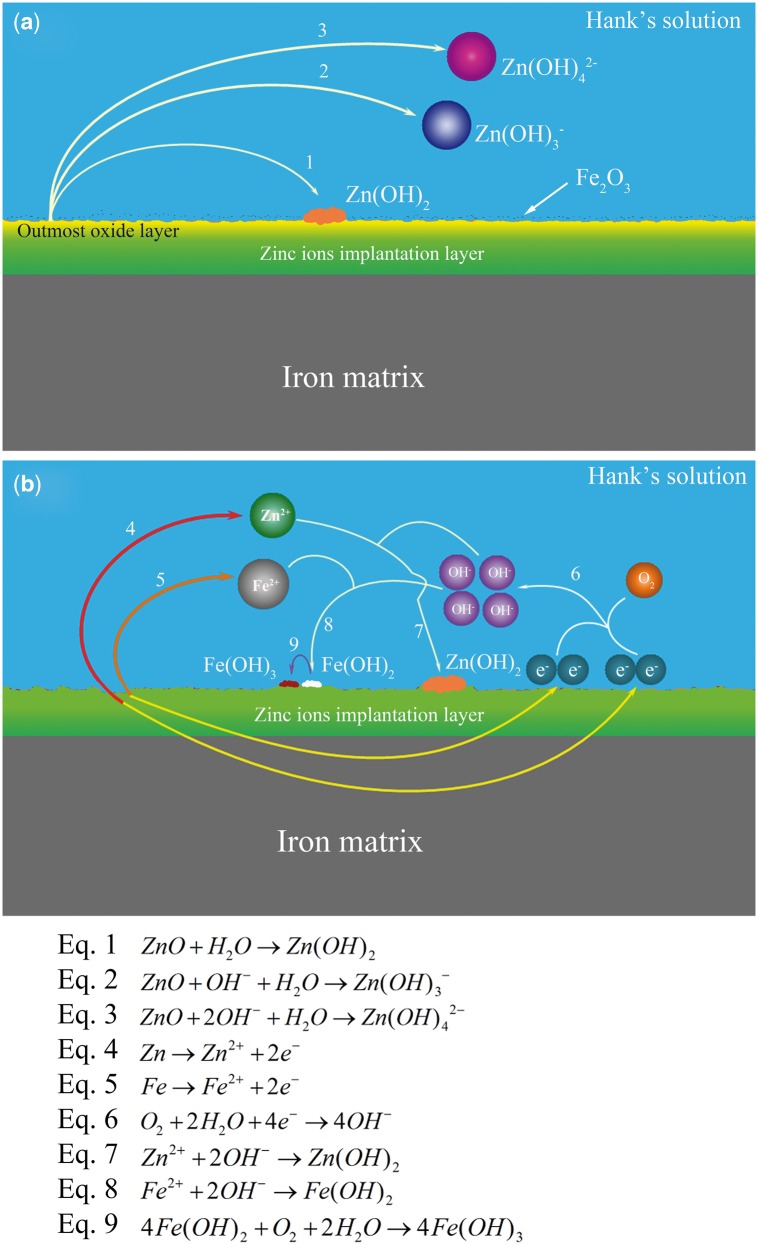
Schematic diagram of the corrosion mechanism for Zn ion implanted pure iron.

The mechanism of Zn ions implanted pure iron is illustrated in Figure 5. According to the results of AES and XPS, the outmost oxide layer was mainly composed of ZnO and Fe_2_O_3_. In the Hank’s solution with dark environment, the reactions in Equations (1)–(3) was caused, and Equation (1) might present the predominant reaction at such pH levels [51], as shown in Figure 5a. With the oxidation of ZnO, the outmost oxidation layer became unstable, then the Fe_2_O_3_ fell off from the surface. After the leave of the outmost oxide layer, the main zinc implantation started being corroded, as shown in Figure 5b. Due to the lower corrosion potential and higher energy, zinc atoms were firstly oxidized, then the structure of iron lattice was collapsed, the released iron atoms with higher energy were oxidized subsequently, showing in Equations (4)–(6). Then, the zinc ions and ferrous ions reacted with Hydroxyl ions to form hydroxide (Equations (7) and (8)). The ferrous hydroxide was easy to be oxidized with the present of dissolved oxygen (Equation (9)).

### Cytotoxicity of zinc ion implanted pure iron

**Figure 6.**
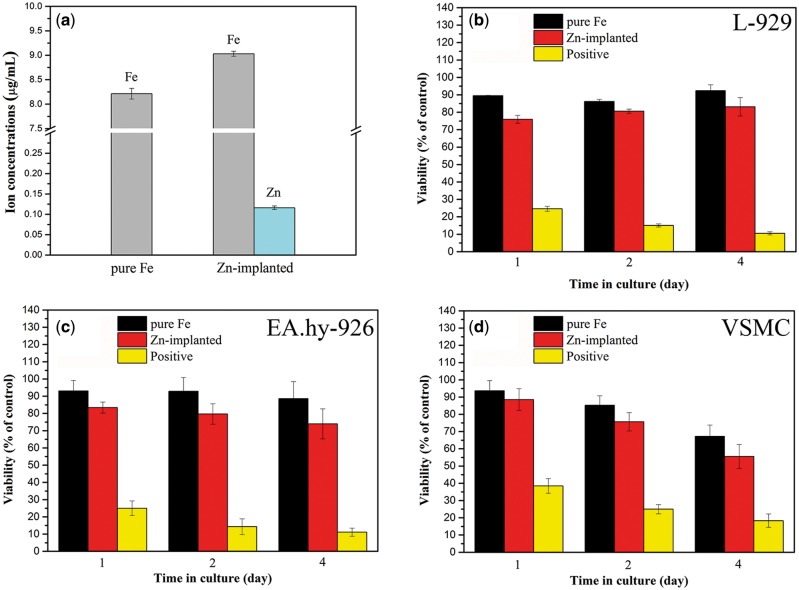
(a) concentrations of ions released from extraction mediums, **(b–d)** are the viabilities of L-929, EA. Hy-926 and VSMC cell lines, respectively.

Figure 6a gives information of the ion concentrations in specimen extractions. The extraction of zinc ion implanted pure iron possessed higher iron ion concentration than pure iron without ion implantation, which matched well with the electrochemical test results and immersion test results. Besides, a small amount of zinc ions were released into the extraction. The cell viabilities of (b) L-929, (c) EA. hy-926 and (d) VSMC are shown in Figure 6b–d. Comparing to pure iron, the zinc ion implantation exhibited negative effects on the viabilities of all tested cell lines. As to the viabilities of L-929 cells and EA.hy-926 cells, the values were maintaining around 80% when incubated in zinc ions implanted pure iron extraction, while those figures kept near 90% in untreated pure iron extraction. With the increase of incubation time, the VSMC viabilities reduced gradually in both zinc ion implanted pure iron and untreated pure iron extractions. After 4 days’ incubation, the viabilities of VSMC decreased to <70%.

Zinc ion has relatively high cytotoxicity [52–54]. The half inhibitory concentration (IC50) of zinc ion to mouse macrophage is about 10 μg·ml^−1^ [55]. Due to the concentration of zinc ions in extraction medium was in a low value of 0.125 μg·ml^−1^, slight toxicity was exhibited to the cells used in these tests.

Pure iron has already exhibited good biocompatibility in numerous *in vitro* and *in vivo* tests [11, 56–59], with an IC50 value of 303 μg·ml^−1^ to murine fibroblasts [60]. When iron ion concentration was lower than 50 μg·ml^−1^, no impact on metabolic activity of endothelial cells was found [61]. According to Figure 6a, the iron ion concentrations in the zinc ion implanted pure iron and untreated pure iron were 9.028 and 8.214 μg·ml^−1^, respectively. Therefore, the high viability of EA.hy-926 can be attributed to the low iron ion concentration in the specimen extractions.

On the other hand, the significantly reduced VSMC viabilities in specimen extractions can be ascribed to the presence of Fe^2+ ^and Fe^3+ ^ions. Schaffer *et al.* [62] found that Fe^2+ ^and Fe^3+ ^ions can inhibit the migration of VSMC. Another research performed by Muller *et al.* [63] demonstrated that Fe^2+ ^can suppress the proliferation of VSMC cells.

### Hemocompatibility of zinc ion implanted pure iron

**Figure 7.**
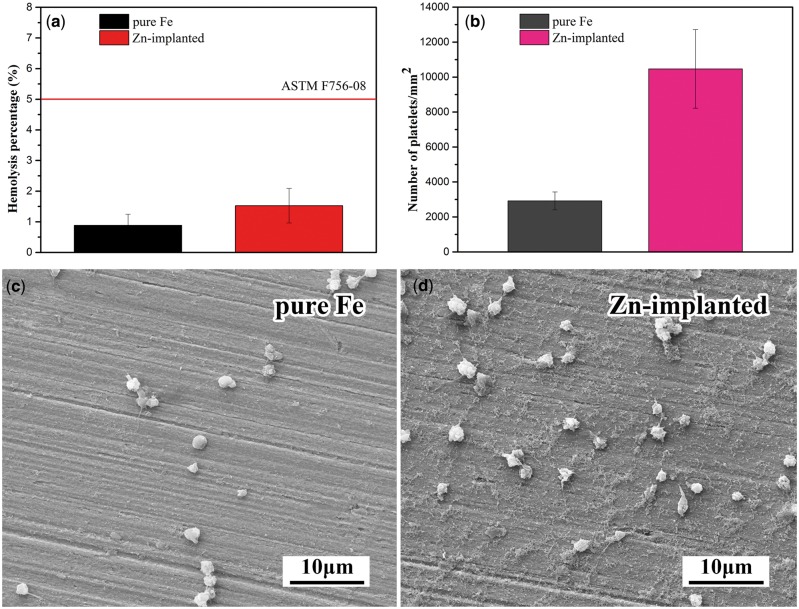
(a) hemolysis test results, **(b)** platelet count results, **(c and d)** are the morphologies of adhered platelets.

Hemolysis is the rupturing of erythrocytes and the release of their contents into surrounding fluid. High hemolysis implies that the material can seriously damage erythrocytes. The standard ASTM F756-08 [64] suggested that the hemolysis of biomaterials in direct contact with blood should be <5%. The hemolysis of zinc ions implanted pure iron was <2% (Fig. 7a), exhibited its slight damaging effect on erythrocytes.

Figure 7b illustrates that zinc ion implantation induced more platelets adhered on the surface of pure iron. The morphologies of adhered platelets are shown in Figure 7c and d. On the surface of zinc ion implanted pure iron, many adhered platelets were activated with pseudopods formed. The activation of platelets is the advance signal of thrombosis [65].

**Figure 8.**
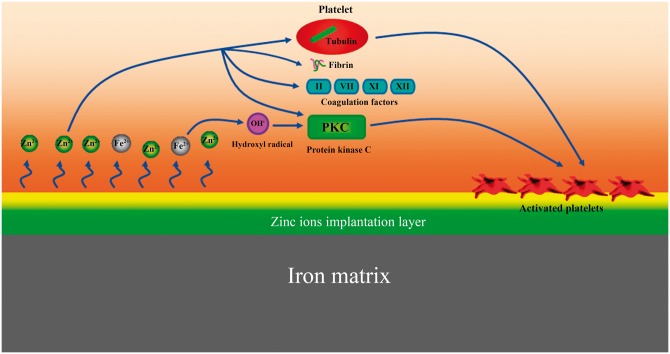
Illustration of the mechanism of platelet activation on the surface of zinc ion implanted pure iron.

The mechanism of platelet activation on the surface of zinc ion implanted pure iron is shown in Figure 8. Due to degradation, Zn^2+ ^and Fe^2+ ^ions were released. Zn^2+ ^ions play a pivotal role in modulating tubulin, which serves a vital role in controlling platelets mobilization, shape change and pseudopod formation [66]. Zn^2+ ^ions can also promote extracellular coagulation and platelet adhesion to one another by modulating fibrin [66]. In addition, the existence of Zn^2+ ^will promote platelet capturing Ca^2+^, then promote the activation of calcium-dependent protein kinase C (PKC), which is an essential enzyme that takes place in platelet activation [67]. Furthermore, Zn^2+ ^can promote the activation of coagulation factors including factors II, VII, XI and XII, which interact with platelets and serves a vital role in coagulation process [67]. On the other hand, the released Fe^2+ ^ions can also contribute to the activation of platelets. Fe^2+ ^ions can catalyze the formation of OH^⋅^, which can` activate PKC, then promote the activation the platelets [68, 69].

## Conclusion

In this work, zinc ions were implanted into pure iron to accelerate degradation rate of pure iron by using MEVVA technology. The chemical compositions and their distribution, *in vitro* degradation behavior, cytotoxicity and hemocompatibility of zinc ion implanted pure iron were systemically investigated. The depth of implanted zinc reached to nearly 60 nm. Zn existed as ZnO in the outer implantation layer, and then gradually transformed to Zn atoms in zero valence with depth. According to the corrosion test results, the implantation of Zn ions could effectively accelerated the corrosion rate of pure iron. In terms of the cytotoxicity, the implanted zinc ions slightly reduced viabilities of cell lines used in this test. On the other hand, zinc ion implanted pure iron displayed slight damaging effect on erythrocytes since their low hemolysis. Nevertheless, the implantation of zinc ions enhanced the adhesion and activation of platelets, which might increase the risk of thrombosis.

*Conflict of interest statement*. None declared.
